# Reply: The clinical and economic benefits of capecitabine and tegafur with uracil in metastatic colorectal cancer

**DOI:** 10.1038/sj.bjc.6603739

**Published:** 2007-04-17

**Authors:** S E Ward

**Affiliations:** 1 School of Health and Related Research, University of Sheffield, Regent Court, 30 Regent Street, Sheffield S1 4DA, UK

**Sir**,

First, the issue of BNF price changes. The prices of the drugs used in the analysis were the current BNF prices at the time the analysis was undertaken. The subsequent price reduction for tegafur-uracil (Uftoral) in March 2006 will, of course, have reduced the cost of this regimen relative to the costs of the other regimens, assuming that the prices of the other drugs have remained unchanged. However, it is not clear from the letter how the reported 59% reduction in drug costs is derived from the 30% reduction in the price of tegafur-uracil capsules shown in the table. Decision makers will need to take into account the cost of the drugs to them at this point in time. The exact cost of these regimens to different institutions may well vary because of negotiated procurement discounts and will, therefore, need to be considered by each institution individually.

The estimated costs of adverse events were subject to significant uncertainty and this was explicitly stated in the article. At the time of the analysis, evidence available from the tegafur-uracil trials did not provide adequate details to allow accurate estimation. The column relating to tegafur-uracil in Table 5 should have been omitted from the paper as these resource use estimates were not ultimately used in the modelling. The hospitalisation data from the table (taken from Ollendorf, 1999) included all nonadministration-related hospitalisations and were considered to be an overestimate of treatment-related adverse events. An alternative assumption was therefore made. Based on evidence that the adverse event profile for tegafur-uracil is equivalent or superior to Mayo in nearly all categories, it was assumed that the resource use rates for treatment-related adverse events would be similar to those incurred with the Mayo regimen. Therefore, for the purposes of our analysis, a conservative assumption was taken that the treatment-related adverse event costs for tegafur-uracil were the same as the treatment-related adverse event costs of the Mayo treatment. The report by Ward *et al* (2003) acknowledged that this may be an overestimate and the same holds true for the BJC paper. Owing to the explicitly stated uncertainties, a sensitivity analysis was presented to demonstrate the impact of removing adverse event costs from the analysis.

The focus of our analysis was to compare oral drugs with intravenous regimens, rather than to seek to differentiate between the oral drugs. We did not highlight the price difference between the oral drugs in the discussion section nor draw any specific conclusions from it. Current NICE guidance recommends that ‘capecitabine or tegafur with uracil (and folinic acid), to be taken by mouth, should be among the first options considered for a person with metastatic colorectal cancer. The choice about which medicine to take should be made jointly by the patient and his or her doctor, and the patient should be informed about the options and the differences between the medicines so that he or she can be fully involved in the decision.’
